# Impressão 3D na Avaliação de Pericardite Constritiva

**DOI:** 10.36660/abc.20220866

**Published:** 2024-03-15

**Authors:** Juliana Cadilho Abrantes, Fernanda Turque, Bernardo Fróes Demier, Daniel Gama Neves, Davi Shunji Yahiro, Tadeu Takao Almodovar Kubo, Leonardo Canale, Claudio Tinoco Mesquita

**Affiliations:** 1 Universidade Federal Fluminense Hospital Universitário Antônio Pedro Niterói RJ Brasil Universidade Federal Fluminense - Hospital Universitário Antônio Pedro, Niterói, RJ – Brasil; 2 Universidade Federal do Rio de Janeiro Rio de Janeiro RJ Brasil Universidade Federal do Rio de Janeiro, Rio de Janeiro, RJ – Brasil; 3 Diagnósticos da América AS Niterói RJ Brasil Diagnósticos da América AS, Niterói, RJ – Brasil; 4 Universidade Federal Fluminense Niterói RJ Brasil Universidade Federal Fluminense, Niterói, RJ – Brasil; 5 Universidade Federal Fluminense Faculdade de Medicina Departamento de Radiologia Niterói RJ Brasil Universidade Federal Fluminense - Faculdade de Medicina - Departamento de Radiologia, Niterói, RJ – Brasil

**Keywords:** Pericardite Constritiva/cirurgia, Pericardiectomia, Imagem Tridimensional/métodos, Educação Médica, Modelos Anatômicos, Planejamento de Assistência ao Paciente

## Introdução

A pericardite constritiva é uma doença pouco frequente e seu diagnóstico muitas vezes é feito tardiamente.^1^ A pericardiectomia radical é o procedimento mais indicado em casos de pericardite constritiva,^2^ entretanto, trata-se de uma cirurgia complexa e demorada. Estes aspectos são potencialmente interessantes para o uso da impressão 3D no planejamento cirúrgico, pois o estudo do modelo impresso pela equipe de saúde permite melhor compreensão da área cirúrgica, obtendo um planejamento mais eficiente.^3 - 5^

Estudos também têm apresentado resultados satisfatórios na educação médica, principalmente em anatomia,^6 , 7^ com relatos de impressão de modelos anatômicos de alta fidelidade, incluindo exemplares com defeitos cardíacos congênitos.^8^ Apesar disso, até o momento, não há relatos do uso da impressão 3D cardíaca no orientação cirúrgica e tampouco no ensino sobre a pericardite constritiva.

Diante disso, a partir do caso de uma paciente com pericardite constritiva grave, avaliamos por meio da impressão 3D a extensão retirada de pericárdio durante a pericardiectomia total. Utilizamos o modelo 3D impresso para ensinar para estudantes de graduação e pós-graduação em ciências cardiovasculares sobre a pericardite constritiva e explicar como é realizado o procedimento de pericardiectomia radical. O objetivo deste relato é, portanto, demonstrar o uso da impressão 3D na educação médica de casos de pericardite constritiva e demonstrar a possibilidade do uso desses modelos no planejamento cirúrgico da pericardiectomia radical.

## Relato de caso

Uma paciente de 51 anos apresentou desconforto abdominal pós-prandial há 5 anos, sendo diagnosticada com cirrose hepática e encaminhada ao hepatologista. Apresentava ao exame, ascite leve e ausência de edema de membros inferiores. Após avaliação do especialista, foi iniciada espironolactona e furosemida. O diagnóstico de cirrose foi refutado, e a paciente foi encaminhada posteriormente a cardiologia devido a calcificação na região do pericárdio. Em 2021, a paciente foi internada por pericardiectomia radical, com circulação extracorpórea de 110 minutos. No pós-operatório a paciente evoluiu com drenagem de grande quantidade de líquido (transudato) por ambos os drenos (Hemotórax direito e esquerdo). Após a retirada dos drenos, foi encaminhada para a enfermaria da Cardiologia, onde permaneceu por 7 dias, recebendo alta para domicílio. A análise histopatológica demonstrou pericardite calcificada. Após a cirurgia foi realizada a impressão 3D do coração e do pericárdio através de imagens de TC realizadas no pré-operatório. O objetivo foi compreender melhor a extensão da doença e o potencial benefício da abordagem cirúrgica.

### Técnica de Impressão 3D

**Figura 1:**
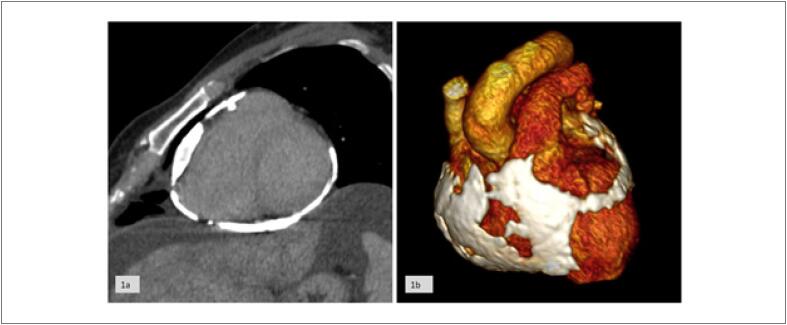
1a) Tomografia Computadorizada do coração com pericárdio calcificado. 1b) Renderização 3D da imagem tomográfica do coração com pericárdio calcificado.

**Figura 2:**
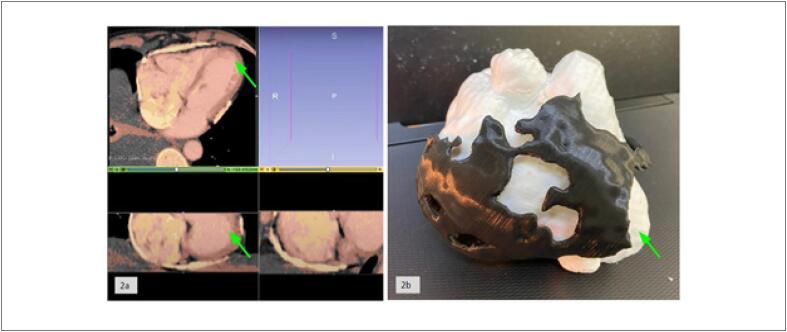
2a) Processo de segmentação da imagem tomográfica e criação do modelo 3D em STL no programa 3D Slicer. Na imagem é possível observar o processo de delimitação do miocárdio em vermelho o do pericárdio calcificado em amarelo. 2b) Modelos impressos em 3D do coração em branco e pericárdio calcificado em preto, acoplados. O ápice do coração está localizado onde aponta a seta verde.

O modelo impresso originou-se de imagens tomográficas do tórax contrastadas, que foram segmentadas selecionando apenas o coração e o pericárdio ( Figura 1a ; Figura 1b ). A imagem foi transformada em um modelo digital salvo no formato *Standard Tesselletion Language (STL)* em um programa de design assistido por computador chamado *3D Slicer* ( Figura 2a ). O procedimento de segmentação durou aproximadamente 2 horas. O modelo foi impresso por meio da tecnologia Fused Deposition Modelling (FDM) que produz peças camada por camada, ao aquecer e extrudar filamentos termoplásticos.^9^ A impressão do coração foi realizada na impressora GTMAX^®^ em baixa qualidade com duração de 20 horas e 55 minutos e o pericárdio foi impresso em uma impressora PRUSA^®^ em ultra qualidade em 21 horas e 12 minutos. Os pesos e tamanhos dos modelos impressos foram, coração 191g com 15% de preenchimento tipo grade com LxPxA: 12,6 × 11,3 × 14,9 cm, pericárdio 94 g com 100% da peça preenchida com LxPxA: 9,7 × 12,2 × 10,2 cm.

Foi possível observar na cirurgia o comprometimento extenso do átrio e do ventrículo direito pela calcificação do pericárdio enquanto o ápice do coração estava livre de calcificação, este padrão de calcificação foi reproduzido fielmente no modelo impresso, sendo possível perceber com exatidão o formato e a espessura do pericárdio calcificado ( Figura 2b ). Durante a segmentação da imagem, notou-se a identificação fidedigna do cálcio presente no pericárdio ao compararmos com a densidade encontrada nas vértebras ( Figura 2a ).

As limitações na qualidade do modelo digital foram a não realização da TC sincronizada ao eletrocardiograma (ECG) e a aquisição em uma fase venosa mais precoce ou uma fase arterial, certamente teriam acarretado uma qualidade da segmentação melhor, o que implicou em menor acurácia do modelo. Entretanto, o fato não comprometeu o entendimento da pericardite constritiva. Desta forma, o modelo não seria indicado em caso de planejamento cirúrgico, no entanto, ele foi utilizado para fins educacionais, demonstrando as estruturas da pericardite constritiva e como foi realizado o procedimento cirúrgico de pericardiectomia total.

A impressão 3D se apresentou apropriado no ensino-aprendizado das características anatomofuncionais da pericardite constritiva para pacientes e profissionais de saúde, e demonstrou a existência da possibilidade do uso da impressão 3D com potencial no planejamento cirúrgico em casos de pericardiectomia.

## Discussão

A utilização da impressão 3D na medicina tem sido alvo de crescente interesse e investigação, oferecendo novas perspectivas para o diagnóstico, planejamento cirúrgico e educação médica.^10^ Neste trabalho, apresentamos a experiência de aplicação da tecnologia de impressão 3D em uma pericardiectomia total, ressaltando seu potencial para fins educacionais e planejamento cirúrgico, mesmo que não tenha sido usada diretamente para o planejamento pré-cirúrgico.

Em relação à dimensão educacional, a impressão 3D possibilita uma visualização tátil e tridimensional das complexas relações anatômicas, tornando-se uma ferramenta poderosa para treinamento prévio, aprimoramento de habilidades técnicas e compreensão mais aprofundada da anatomia cardíaca.^8^ A utilização da impressão 3D como recurso educacional pôde promover a colaboração interdisciplinar, permitindo que cardiologistas, cirurgiões e radiologistas trabalhassem de forma integrada na compreensão dos detalhes anatômicos e no aprimoramento das estratégias de abordagem cirúrgica. Além de ser valiosa para alunos de graduação da área da saúde para entenderem com mais clareza os aspectos anatomopatológicos da doença.^8^ A revisão de Ford e Minshall^6^ relata que essa tecnologia já está sendo usada até mesmo para ensino de pessoas com deficiência visual ou cegas em outras áreas do conhecimento, como por exemplo em matemática, história ou geociência.^6^

**Figura 3:**
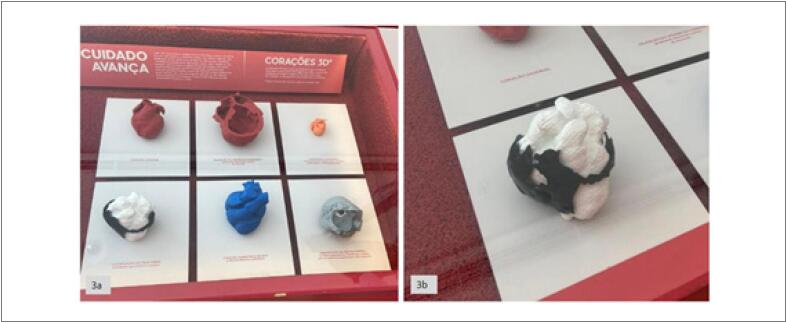
– 3a) Modelos em exposição no renomado Museu do Amanhã na exposição “Coração S2, Pulso da Vida”. 3b) Modelo do estudo que foi apresentado no Museu do Amanhã na exposição “Coração S2, Pulso da Vida.

Outra faceta intrigante da aplicação da impressão 3D nesse cenário foi sua utilização para o contato com o público em geral para a visualização das várias formas que o coração humano pode assumir.^11^ O modelo impresso foi apresentado em uma exposição no renomado Museu do Amanhã na exposição “Coração S2, Pulso da Vida” ( Figura 3 ). Essa iniciativa exemplifica como a impressão 3D pode transcender as fronteiras da medicina puramente técnica e se insere em contextos que ampliam o alcance da ciência médica para a sociedade em geral. A exposição do coração impresso proporcionou uma oportunidade única para a população apreciar a complexidade da anatomia cardíaca. Essa abordagem contribui ainda mais para a humanização da medicina, aproximando as pessoas do coração como símbolo de vida e vitalidade, o que ajuda na comunicação com o paciente.^9^ Além disso, a exposição serviu como um veículo poderoso para a divulgação científica, ampliando a atenção do público sobre a importância de doenças cardiovasculares e as conquistas da medicina moderna.

Embora a impressão 3D não tenha sido utilizada diretamente no planejamento cirúrgico da pericardiectomia total, seus benefícios não devem ser subestimados. A tecnologia de impressão 3D continua a evoluir, e sua aplicação educacional abre caminho para futuras investigações e possíveis aplicações diretas no planejamento cirúrgico de intervenções complexas.

## Conclusão

A integração da impressão 3D em cenários de saúde representa uma mudança de paradigma. Este estudo sugere que a impressão 3D pode ter um papel significativo no ensino das características anatomofuncionais da pericardite constritiva e na orientação sobre como é realizado o procedimento de pericardiectomia total.
