# Supplementary material for: Impressão 3D na Avaliação de Pericardite Constritiva
Source: Arq Bras Cardiol. 2024 Mar 15;121(2):e20220866. [Article in Portuguese] doi: 10.36660/abc.20220866 (PMC12092021; doi:10.36660/abc.20220866)
Supplement: Translation

# Translation: 3D Printing in Constrictive Pericarditis Assessment

**Authors:** Juliana Cadilho Abrantes, Fernanda Turque, Bernardo Fróes Demier, Daniel Gama Neves, Davi Shunji Yahiro, Tadeu Takao Almodovar Kubo, Leonardo Canale, Claudio Tinoco Mesquita

**Affiliations:** 1 Niterói RJ Brazil Universidade Federal Fluminense - Hospital Universitário Antônio Pedro, Niterói, RJ – Brazil; 2 Rio de Janeiro RJ Brazil Universidade Federal do Rio de Janeiro, Rio de Janeiro, RJ – Brazil; 3 Niterói RJ Brazil Diagnósticos da América AS, Niterói, RJ – Brazil; 4 Niterói RJ Brazil Universidade Federal Fluminense, Niterói, RJ – Brazil; 5 Niterói RJ Brazil Universidade Federal Fluminense - Faculdade de Medicina - Departamento de Radiologia, Niterói, RJ – Brazil

**Keywords:** Pericarditis Constrictive/surgery, Pericardiectomy/surgery, Imaging, Threedimensional/methods, Education Medical, Patient Care Planning, Models Anatomic, Patient Caare Planning

## Introduction

Constrictive pericarditis is a rare disease, often diagnosed belatedly.^1^Radical pericardiectomy is the recommended procedure in cases of constrictive pericarditis,^2^although it is a complex and time-consuming surgery. These aspects make 3D printing potentially valuable in surgical planning, allowing the healthcare team to study a printed model for better comprehension of the surgical area and more efficient planning.^3 - 5^

Studies have also shown satisfactory results in medical education, particularly in anatomy,^6 , 7^with reports of high-fidelity anatomical models, including those with congenital heart defects.^8^However, until now, there have been no reports of using 3D cardiac printing for surgical guidance or teaching about constrictive pericarditis.

Therefore, based on a severe case of constrictive pericarditis in a patient, we evaluated the extent of pericardial removal during total pericardiectomy using 3D printing. We used the 3D printed model to educate undergraduate and postgraduate cardiovascular science students about constrictive pericarditis and to explain how the radical pericardiectomy procedure is performed. This report aims to demonstrate the use of 3D printing in medical education for cases of constrictive pericarditis and to show the potential use of these models in the surgical planning of radical pericardiectomy.

## Case Report

A 51-year-old patient presented with postprandial abdominal discomfort for 5 years, initially diagnosed with liver cirrhosis and referred to a hepatologist. She had mild ascites and no lower limb edema on examination. After evaluation by the specialist, spironolactone and furosemide were initiated. The diagnosis of cirrhosis was refuted, and subsequently, due to pericardial calcification, the patient was referred to cardiology. In 2021, the patient underwent radical pericardiectomy, with 110 minutes of extracorporeal circulation. Postoperatively, the patient had significant fluid drainage (transudate) from both drains (right and left hemithorax). After drain removal, she was transferred to the Cardiology ward, where she stayed for 7 days before being discharged home. Histopathological analysis demonstrated calcified pericarditis. After surgery, a 3D printing of the heart and pericardium was performed using preoperative CT images to understand better the extent of the disease and the potential benefit of the surgical approach.

### 3D Printing

The printed model originated from contrasted thoracic tomographic images that were segmented to select only the heart and pericardium ( Figure 1a ; Figure 1b ). The image was transformed into a digital model saved in Standard Tessellation Language (STL) format using a computer-aided design program called 3D Slicer ( Figure 2a ). The segmentation process took approximately 2 hours. The model was printed using Fused Deposition Modelling (FDM) technology, producing layer-by-layer pieces by heating and extruding thermoplastic filaments.^9^Printing of the heart took place on a GTMAX^®^printer at low quality, lasting 20 hours and 55 minutes, and the pericardium was printed on a PRUSA^®^printer at ultra-quality for 21 hours and 12 minutes. The weights and sizes of the printed models were: heart 191g with 15% grid-type filling at 12.6 × 11.3 × 14.9 cm, pericardium 94g fully filled at 9.7 × 12.2 × 10.2 cm.

**Figure 1:** 1a) Computed Tomography of the heart with calcified pericardium. 1b) 3D rendering of the tomographic image of the heart with calcified pericardium.

During surgery, extensive involvement of the right atrium and ventricle by pericardial calcification was observed, while the heart's apex was free of calcification. This pattern of calcification was faithfully reproduced in the printed model, accurately displaying the shape and thickness of the calcified pericardium ( Figure 2b ). During image segmentation, the accurate identification of calcium in the pericardium was noted by comparing it to the density found in the vertebrae ( Figure 2a ). Limitations in the digital model's quality were the lack of synchronized CT with the electrocardiogram (ECG) and the acquisition in an earlier venous phase or an arterial phase. Certainly, a better phase of acquisition would have resulted in improved segmentation quality, enhancing the model's accuracy. However, this limitation did not compromise the understanding of constrictive pericarditis. Thus, while the model was not recommended for surgical planning, it was used for educational purposes, demonstrating the structures of constrictive pericarditis and how the total pericardiectomy surgical procedure was performed.

**Figure 2:** 2a) Image segmentation process and creation of the 3D model in STL format in the 3D Slicer program. In the image, it is possible to observe the delimitation process of the myocardium in red and the calcified pericardium in yellow. 2b) 3D printed models of the heart in white and calcified pericardium in black, joined. The apex of the heart is located where the green arrow points.

3D printing proved suitable for teaching and learning the anatomofunctional characteristics of constrictive pericarditis for both patients and healthcare professionals, demonstrating the potential use of 3D printing in the surgical planning of pericardiectomy.

## Discussion

The use of 3D printing in medicine has garnered increasing interest and investigation, offering new perspectives for diagnosis, surgical planning, and medical education.^10^In this work, we present the experience of applying 3D printing technology in a total pericardiectomy, highlighting its potential for educational and surgical planning purposes, even though it was not directly used for pre-surgical planning.

Regarding its educational dimension, 3D printing allows tactile and three-dimensional visualization of complex anatomical relationships, becoming a powerful tool for pre-training, technical skill enhancement, and a deeper understanding of cardiac anatomy.^8^Using 3D printing as an educational resource can promote interdisciplinary collaboration, allowing cardiologists, surgeons, and radiologists to work together to understand anatomical details and improve surgical approach strategies. It is also valuable for undergraduate health science students to gain clearer insights into anatomopathological aspects of the disease.^8^The review by Ford and Minshall^6^reports that this technology is already being used even for teaching visually impaired or blind individuals in other areas of knowledge, such as mathematics, history, and geoscience.^6^

Another intriguing aspect of 3D printing's application in this scenario was its use for public engagement to visualize the various shapes the human heart can assume.^11^The printed model was exhibited in the renowned Museum of Tomorrow in the “Heart S2, Pulse of Life” exhibition ( Figure 3 ). This initiative exemplifies how 3D printing can transcend the boundaries of purely technical medicine and fit into contexts that broaden the reach of medical science to society at large. The display of the printed heart provided a unique opportunity for the public to appreciate the complexity of cardiac anatomy. This approach further contributes to humanizing medicine, bringing people closer to the heart as a symbol of life and vitality, and aiding in communication with patients.^9^Additionally, the exhibition served as a powerful vehicle for scientific outreach, increasing public attention to the importance of cardiovascular diseases and the achievements of modern medicine.

**Figure 3:** 3a) Models on display at the renowned Museum of Tomorrow in the exhibition “Heart S2, Pulse of Life”. 3b) Model from the study presented at the Museum of Tomorrow in the exhibition “Heart S2, Pulse of Life”.

Although 3D printing was not directly used in the surgical planning of total pericardiectomy, its benefits should not be underestimated. 3D printing technology continues to evolve, and its educational application paves the way for future investigations and potential direct applications in the surgical planning of complex interventions.

## Conclusion

The integration of 3D printing into healthcare settings represents a paradigm shift. This study suggests that 3D printing can play a significant role in teaching the anatomofunctional characteristics of constrictive pericarditis and in guiding how the total pericardiectomy procedure is performed.
